# Impact of Influenza on Pneumococcal Vaccine Effectiveness during *Streptococcus pneumoniae* Infection in Aged Murine Lung

**DOI:** 10.3390/vaccines8020298

**Published:** 2020-06-11

**Authors:** Ermias Jirru, Stefi Lee, Rebecca Harris, Jianjun Yang, Soo Jung Cho, Heather Stout-Delgado

**Affiliations:** Weill Cornell Medicine, Division of Pulmonary and Critical Care, New York, NY 10022, USA; ekj9002@med.cornell.edu (E.J.); sfl9003@med.cornell.edu (S.L.); rmh2004@med.cornell.edu (R.H.); jiy2017@med.cornell.edu (J.Y.); sjc9006@med.cornell.edu (S.J.C.)

**Keywords:** aging, viral immune imprinting, influenza, *Streptococcus pneumoniae*, vaccine effectiveness

## Abstract

Changes in innate and adaptive immune responses caused by viral imprinting can have a significant direct or indirect influence on secondary infections and vaccine responses. The purpose of our current study was to investigate the role of immune imprinting by influenza on pneumococcal vaccine effectiveness during *Streptococcus pneumoniae* infection in the aged murine lung. Aged adult (18 months) mice were vaccinated with the pneumococcal polyvalent vaccine Pneumovax (5 mg/mouse). Fourteen days post vaccination, mice were instilled with PBS or influenza A/PR8/34 virus (3.5 × 10^2^ PFU). Control and influenza-infected mice were instilled with PBS or *S. pneumoniae* (1 × 10^3^ CFU, ATCC 6303) on day 7 of infection and antibacterial immune responses were assessed in the lung. Our results illustrate that, in response to a primary influenza infection, there was diminished bacterial clearance and heightened production of pro-inflammatory cytokines, such as IL6 and IL1β. Vaccination with Pneumovax decreased pro-inflammatory cytokine production by modulating NFҡB expression; however, these responses were significantly diminished after influenza infection. Taken together, the data in our current study illustrate that immune imprinting by influenza diminishes pneumococcal vaccine efficacy and, thereby, may contribute to increased susceptibility of older persons to a secondary infection with *S. pneumoniae*.

## 1. Introduction

People worldwide are living longer, and it is estimated that, by 2050, the proportion of the world’s population over 60 years of age will nearly double. Natural lung aging is associated with molecular and physiological changes that cause alterations in lung function, diminished pulmonary remodeling and regenerative capacity, and increased susceptibility to acute and chronic lung diseases. In addition, diminished immune function and age-associated changes in primary vaccine responses contribute to weakened long-lasting antibody responses to pathogenic stimuli. As the aging population rapidly grows, it is essential to examine how alterations in cellular function and cell-to-cell interactions of pulmonary resident cells and systemic immune cells contribute to a higher risk of increased susceptibility to infection.

Influenza epidemics still remain a leading cause of morbidity and mortality worldwide, with the highest incidence of hospitalization and death occurring in persons >65 years of age. Age-associated alterations in immune surveillance—specifically, decreased innate and adaptive immune responses and dysfunctional immune regulation—can be responsible for increased lung pathology to infectious stimuli [1,2,3,4,5,6]. A primary influenza infection, through virally imprinting on immune responsiveness, can have a significant direct or indirect influence on secondary infections and vaccine efficacy [7,8]. Bacteria, especially *Streptococcus pneumoniae*, are the most common pathogens that commonly cause pneumonia in the elderly [2]. Upregulation of epithelial cell surface receptors in response to chronic inflammation can increase bacterial adhesion and accumulation in the aged lung and correlate with an age-associated increased in host susceptibility to pneumonia [2,9]. Depressed clearance mechanisms, such as cough, oral and mucociliary clearance, and swallowing disorders, can also increase susceptibility [10,11,12,13].

Immune imprinting by a viral infection has the potential to influence host responsiveness to subsequent infections. Many studies have illustrated the role of a primary influenza infection in modulating host responses to a subsequent strain of influenza [7,8,14,15,16,17,18]. It has been suggested that negative antigenic interaction, or pre-immunity from a person’s first influenza strain, due to changes in the memory immune responses, may have deleterious effects on clinical outcomes during a secondary infection or host vaccine memory responsiveness [19]. The timing between influenza infection and post-viral pneumococcal infection as well as the dose of bacteria administered can greatly impact the host outcomes of a secondary infection [17,20,21,22,23]. Specifically, the initiation of influenza-virus-mediated lung inflammation has been shown to directly correlate with bacterial growth, and high dosages of bacteria have been associated with increased mortality during secondary infection [17,20,21,22,23,24]. Previous work has illustrated that, as early as day 3 post influenza, bacterial clearance from the lung was significantly altered [25]. Progression of influenza and viral-mediated changes in the immune response contribute to increased virus-mediated damage to the lung and resulted in greater susceptibility to a secondary pneumococcal infection [18,24,26,27,28,29]. Further, recent work has demonstrated that, in the absence of influenza, young adult mice immunized with pneumococcal-specific vaccines are highly efficient in pulmonary clearance of the bacteria [30,31]. In the presence of influenza, vaccine protection was dramatically reduced [17,30,31]. Vaccine variations, such as immunization with pneumococcal surface protein A (PspA), engineered live-attenuated vaccines, and dual vaccine/cytokine administration, have been shown to fully protect influenza-infected mice against a physiologically relevant dose of *S. pneumoniae* [31,32,33]. Taken together, the cascade of innate and adaptive immune responses to an immune imprinting event can greatly impact host susceptibility to secondary infection [7].

For adults >65 years of age, the 23-valent pneumococcal polysaccharide vaccine (PPV23) Pneumovax and the 13-valent pneumococcal conjugate vaccine (PCV13) Prevnar are two vaccines available for protection against pneumococcal infections. While there have been conflicts in pneumococcal vaccine effectiveness, a recent meta-analysis illustrated that Pneumovax exhibited a weak protective effect on all-cause pneumonia among immunocompetent adults and persons over 65 years of age as well as high-risk persons (19–64 years of age) [34]. While multiple studies have illustrated that vaccination in persons >60 years with Prevnar can result in improved immunogenicity against multiple *S. pneumoniae* serotypes, these antibody titers were found to decline after a year and were similar to titers observed post Pneumovax vaccination [35,36,37,38]. In addition, combined administration of Prevnar prior to Pneumovax can elicit a greater immune response than multiple dosages of Prevnar, which only demonstrated a modest increase [37,39]. As recent work has illustrated differential efficacy of Prevnar vaccination in modulating the immune responses of adult mice to post-influenza infection with a serotype 3 strain of *Streptococcus pneumoniae*, we chose to examine the impact of influenza infection on Pneumovax responses during a secondary bacterial infection [30,40]. Specifically, the purpose of our current study was to investigate the role of immune imprinting by influenza on pneumococcal vaccine effectiveness during *S. pneumoniae* infection in the aged murine lung. Aged adult (18 months) mice were vaccinated with the pneumococcal polyvalent vaccine Pneumovax (5 mg/mouse). Mice were instilled with PBS or influenza A/PR8/34 virus (3.5 × 10^2^ PFU) 14 days post vaccination. On day 7, control and influenza-infected mice were instilled with PBS or *S. pneumoniae* (1 × 10^2^ CFU, ATCC 6303) and antibacterial immune responses were assessed in the lung. Our results illustrate that, in response to a primary influenza infection, there was diminished bacterial clearance and heightened production of pro-inflammatory cytokines, such as IL6 and IL1β. Vaccination with Pneumovax decreased pro-inflammatory cytokine production by modulating NF-ҡB expression; however, these responses were significantly diminished after influenza infection. Taken together, the data in our current study illustrate that immune imprinting by influenza diminishes pneumococcal vaccine efficacy and, thereby, may contribute to the increased susceptibility of older persons to a secondary infection with *S. pneumoniae*.

## 2. Materials and Methods

### 2.1. Mice

Young adult (3–4 months) and aged adult (18–20 months of age) male and female BALB/c mice were purchased from the NIA rodent facility (Charles River Laboratories). Upon receipt, mice were handled under identical husbandry conditions and fed certified commercial feed. Body weights were measured daily, and mice were humanely euthanized if they lost more than 15% of their starting body weight. The IACUC at Weill Cornell Medicine approved the use of animals in this study, and methods were carried out in accordance with the relevant guidelines and regulations. No animals were used in the study if there was evidence of skin lesions, weight loss, or lymphadenopathy.

### 2.2. Influenza (A/Puerto Rico/8/1934, H1N1)

Influenza viral stock (material #: 10100374, batch #: 4XP170531, EID_50_ per ml: 10^10.3^) was purchased from Charles River Laboratories (Norwich, CT, USA). All mice were anesthetized with isoflurane (5% for induction and 2% for maintenance) prior to intranasal instillation with 3.5 × 10^2^ PFU of influenza (50-µL volume in PBS). TCID_50_ was calculated using the Viral ToxGlo Assay (Promega, Madison, WI, USA). Briefly, BAL was diluted in 3.16-fold serial dilutions and plated for 24–48 h on >80% confluent MDCK cells. Upon visualization of cytopathic effect, ATP detection reagent was added and luminescence was measured. Values were calculated by plotting net relative luminescence unit (RLU) values after subtracting average blank wells against viral dilution. The TCID50 value is the reciprocal of the dilution that produced a 50% decline in ATP levels compared to untreated controls. Validated regression analysis was performed using GraphPad Prism (GraphPad Software, San Diego, CA, USA).

### 2.3. Streptococcus Pneumoniae

*S. pneumoniae* (6303, ATCC Manassas, VA, USA) was grown on 10% sheep blood agar plates (BD Biosciences, San Jose, CA, USA) overnight at 37 °C, 5% CO^2^. Colonies were collected on an inoculating loop and added to 10 mL of THY (Todd Hewitt Broth + 5% yeast extract) in a 125-mL polystyrene flask. Flasks were incubated at 37 °C, 5% CO2 and 200 rpm for 3–4 h. Colony-forming units were quantified by dilution of samples in PBS, and titers were determined by colony counts × dilution. All mice were intranasally instilled with 1 × 10^3^ colony-forming (CFU) units of *S. pneumoniae* (50-μL vol in PBS) after anesthetization with isoflurane (5% for induction and 2% for maintenance).

### 2.4. In Vivo Procedures and Tissue Collection

Pneumovax vaccination: Pneumovax (PPV-23) vaccine was purchased from Henry Schein Medical (Newburgh, NY, USA). Mice were vaccinated with 100 mL of vaccine (5 mg) via subcutaneous injection on day 0. Bronchoalveolar lavage (BAL): BAL was collected using previously published methods [41]. Briefly, 0.8 mL of PBS was slowly injected and aspirated 4 times prior to saving the recovered lavage fluid on ice. Lavage was clarified at 1500 rpm for 10 min at 4 °C. Lung tissue collection: at selected time points of infection, lung tissue was collected from control and influenza-infected young and aged adult mice. Tissue was snap frozen or placed into Allprotect (Qiagen, Germantown, MD, USA) for future analysis. Histology: mice were euthanized and right lung tissue was collected for downstream analysis. To maintain architecture, the left lung was distended with 1% low-melting agarose and placed into cold formalin [42]. Tissue samples were processed and H&E stained by the Translational Research Program at WCM Pathology and Laboratory of Medicine. Images were scanned using the EVOS FL Auto Imaging System (ThermoFisher Scientific, Fair Lawn, NJ, USA). For all animal experiments, we used 5–10 mice per group, and experiments were repeated at least three times.

### 2.5. ELISA

Culture supernatants, lung homogenates, and BAL were analyzed for IL1β and IL6 production using ELISA kits purchased from ThermoFisher Scientific. Protein levels in BAL were calculated using the BioRad protein assay (BioRad, Hercules, CA, USA) per manufacturer’s instructions. IgM and IgG antibody ELISAs were performed similarly to previously described methods [33]. Briefly, serum from PBS and Pneumovax-vaccinated mice was serially diluted in PBS + 1% BSA and incubated on ELISA plates precoated with 50-mL of 15 mg/mL solution of unconjugated PPS3 (ATCC) in 0.05 M carbonate–bicarbonate buffer, pH 9.6 (Sigma Aldrich, St. Louis, MO, USA). After overnight incubation, the plates were washed, and specific antibody titers were detected with anti-mouse IgM or IgG secondary antibodies (Ready-SET Go, mouse IgM and IgG kits, ThermoFisher Scientific).

### 2.6. RNA Purification and Real Time PCR

RNA samples were extracted using the automated Maxwell RNA extraction protocol (Madison, WI). Samples were quantified and A_260/280_ ratios were recorded. Samples were reverse-transcribed using the First Stand Synthesis Kit and quantified using QuantiTect Primer Assays, and RT^2^ Profiler^TM^ Assays (Mouse Antibacterial Response PAMM-148Z) were used to assess gene expression (Qiagen). All reactions were performed in triplicate. Relative levels of messenger RNA (mRNA) were calculated by the comparative cycle threshold method, and either β-Actin or β2 M mRNA levels were used as the invariant control for each sample. Fold change expression values were assessed by Ingenuity Pathway Analysis (IPA) (Qiagen).

### 2.7. Statistical Analysis

Survival analysis between groups was calculated using the Mantel–Cox test. Comparison of groups was performed using a two-tailed *t*-test and comparisons between groups were verified by one-way ANOVA. All samples were independent and contained the same sample size for analysis. All data were analyzed using GraphPad Prism. Statistical significance was considered as * *p* < 0.05, ** *p* < 0.01, *** *p* < 0.001, and **** *p* < 0.0001.

### 2.8. Data Availability

Most data generated during this study are included in this published article. The datasets generated during and/or analyzed during the current study are available from the corresponding author on reasonable request.

## 3. Results

As viral imprinting has the potential to influence susceptibility to secondary infections, the purpose of our current study was to determine if host immune responses initiated during influenza can impact pneumococcal vaccine efficacy against a secondary bacterial infection.

### 3.1. Impact of Primary Influenza Infection on Susceptibility of Aged Adult Mice to S. pneumoniae

**Figure 1:**
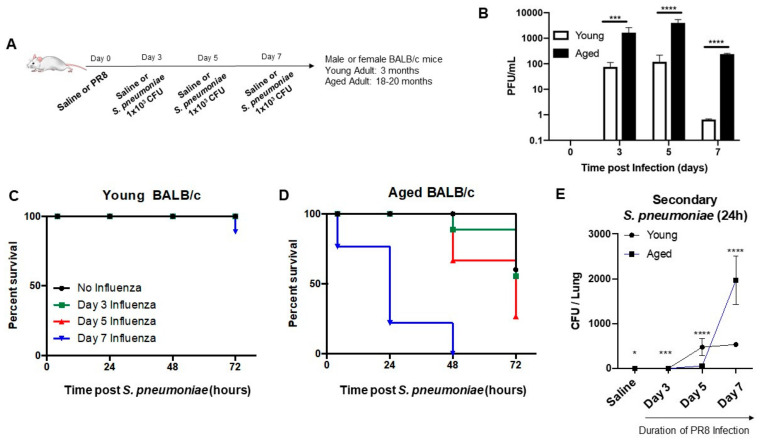
*Impact of primary influenza infection on susceptibility of aged adult mice to S. pneumoniae*. (**A**) Young (3 months) and aged adult (18–20 months) mice were intranasally instilled with PBS or influenza (A/Puerto Rico/8/1934, H1N1) prior to secondary intranasal instillation with *S. pneumoniae* (1 × 10^3^ CFU) on day 3, 5, or 7 of influenza infection. (**B**) Viral titers were quantified in young and aged adult BAL samples collected at select time points post infection by serial dilution. (**C**,**D**) Survival was assessed in young and aged adults at 24–72 h post secondary *S. pneumoniae* instillation. Black line: PBS/no influenza, green line: *S. pneumoniae* administered at day 3 of influenza, red line: *S. pneumoniae* administered at day 5 of influenza, blue line: *S. pneumoniae* administered at day 7 of influenza. (**E**) Bacterial titers in BAL collected at 24 h post secondary *S. pneumoniae* infection were quantified in young and aged adult lung. Student’s *t*-test: * *p* < 0.05, *** *p* < 0.001, and **** *p* < 0.0001. Similar results were obtained from at least three independent experiments, with N = 5 per group. Data are expressed as the mean ± SD.

To understand how a primary infection with influenza might modulate host susceptibility to *S. pneumoniae*, we infected young (3 months) and aged adult (18–20 months) mice with influenza (A/Puerto Rico/8/1934, H1N1) prior to secondary instillation with *S. pneumoniae* on day 3, 5, or 7 of influenza infection (Figure 1A). To examine the impact of viral titer on host susceptibility to secondary *S. pneumoniae*, we examined viremia in BAL samples collected from young and aged adult mice at select time points during influenza. When compared to young, there were significantly increased viral titers detected in aged adult lung at all time points of infection (Figure 1B). In contrast to young adult mice, there was a significant increase in lethality in aged mice, which corresponded with the duration of influenza infection (Figure 1C,D). Specifically, there was increased lethality in aged mice in response to a secondary *S. pneumoniae* infection on day 3 of influenza (Figure 1D). Lethality continued to increase, with aged adult mice becoming highly susceptible to secondary *S. pneumoniae* infections at day 7 post influenza (Figure 1D). Examination of bacterial titers illustrated that there were significantly higher levels of *S. pneumoniae* present in BAL isolated from uninfected and influenza-infected (day 3 post infection) aged mice (Figure 1E). Interestingly, when compared to aged adults, at day 5 post influenza there was a significant increase in bacterial titers in young adult lung (Figure 1E). However, by day 7 of influenza infection, there was an abundance of *S. pneumoniae* present in aged adult lung, with levels significantly higher than in young (Figure 1E). Taken together, these results illustrate that a primary infection with influenza can directly impact host susceptibility to *S. pneumoniae*, with increased bacterial titers and lethality in aged adult mice.

### 3.2. Increased Changes in Histopathology in Aged Adult Murine Lung During Primary Influenza and Secondary S. pneumoniae Infection

**Figure 2:**
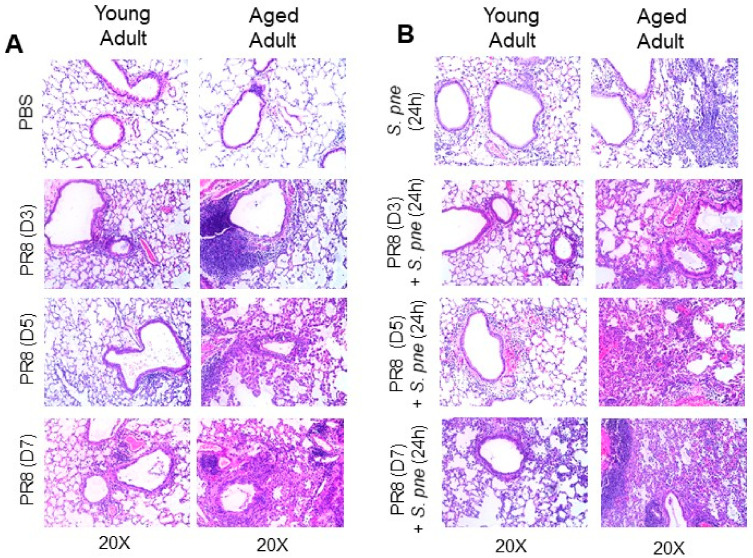
*Increased changes in histopathology in aged adult murine lung during primary influenza and secondary S. pneumoniae infection*. Young (3 months) and aged adult (18–20 months) mice were intranasally instilled with PBS or influenza (A/Puerto Rico/8/1934, H1N1) prior to secondary intranasal instillation with *S. pneumoniae* (1 × 10^3^ CFU) on day 3, 5, or 7 of influenza infection. Lung tissue was collected at select time points post infection and lung histology was examined by H&E staining. (**A**) Representative lung histology in young and aged adult lung in response to influenza and (**B**) *S. pneumoniae* at select time points post influenza infection. Similar results were obtained from at least three independent experiments, with N = 5 per group.

To expand our initial findings, we next examined the histopathological changes that occurred in young and aged adult lung in response to primary influenza and the impact of these alterations on host responses to secondary *S. pneumoniae*. In response to influenza, there was cellular infiltration in both young adult and aged adult murine lung, with marked levels of immune cells recruited to the aged lung (Figure 2A). As the course of influenza infection progressed, there was a continued increase in inflammation and cellular recruitment in the aged lung (Figure 2A). In response to primary and secondary *S. pneumoniae*, there was also a marked increase in inflammatory damage to the alveolar capillary barrier, resulting in increased permeability and intra-alveolar edema in the aged lung (Figure 2B). In sum, in response to influenza, there are increased changes in histopathology in aged adult murine lung, with increased inflammation, cellular infiltration, and inflammatory damage occurring post viral *S. pneumoniae*.

### 3.3. Impact of Influenza on Pneumococcal Vaccine Effictiveness During S. pneumoniae Infection in Aged Lung

**Figure 3:**
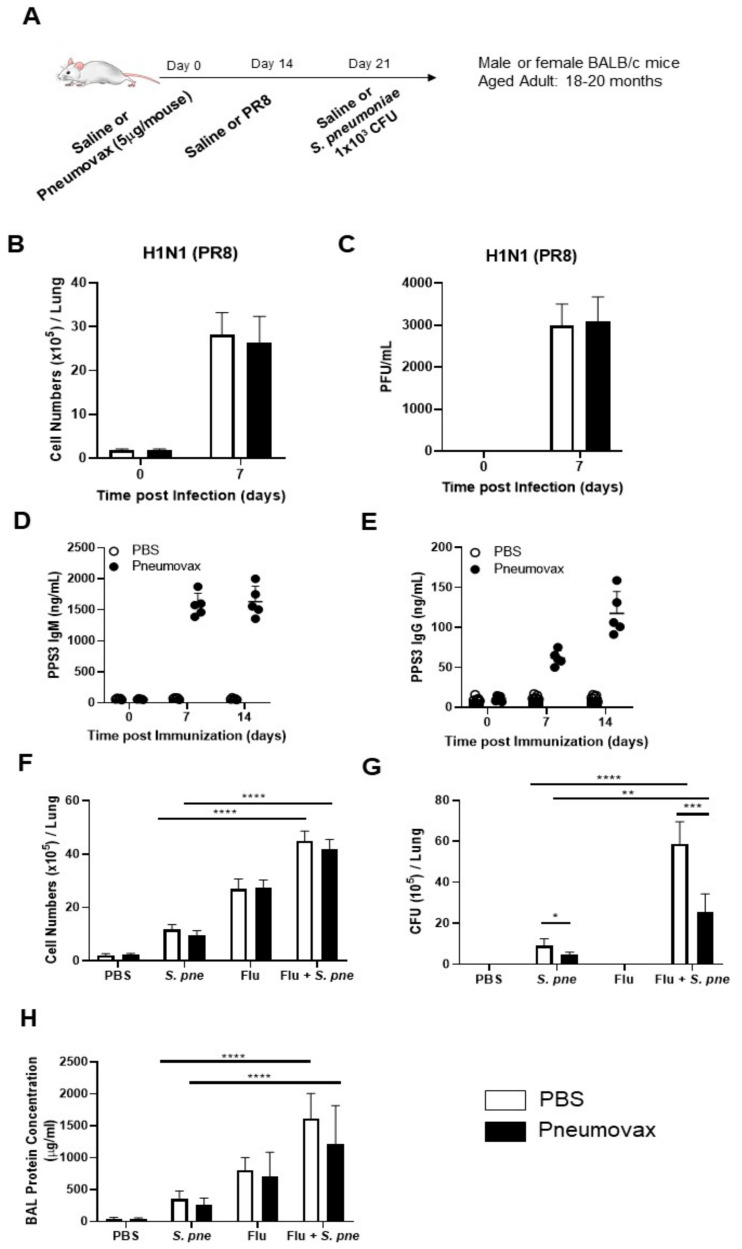
*Impact of influenza on pneumococcal vaccine effectiveness during S. pneumoniae infection in aged lung*. (**A**) Aged adult (18–20 months) mice received a subcutaneous injection of PBS or Pneumovax (5 mg/mouse) on day 0. On day 14 post vaccination, mice were intranasally instilled with PBS or influenza (A/Puerto Rico/8/1934, H1N1) prior to secondary intranasal instillation with *S. pneumoniae* (1 × 10^3^ CFU) on day 7 post influenza. (**B**) Total cell counts and (**C**) viral titer were assessed in BAL collected from PBS- and Pneumovax-treated mice on day of influenza. (**D**) IgM and (**E**) IgG PPS3 antibody levels were assessed in serum collected on day 7 and 14 post vaccination. (**F**) Total cell counts, (**G**) bacterial titers, and (**H**) protein concentration in BAL collected at 24 h post-secondary *S. pneumoniae* infection were quantified in aged adult lung. Student’s *t*-test: * *p* < 0.05, ** *p* < 0.01, *** *p* < 0.001, and **** *p* < 0.0001. Similar results were obtained from at least three independent experiments, with N = 5 per group. Data are expressed as the mean ± SD.

Based on these findings, we next investigated the impact of influenza on pneumococcal vaccine efficacy during *S. pneumoniae* infection in aged lung. Specifically, aged adult mice were vaccinated with Pneumovax (5 mg/mouse) on day 0 (Figure 3A). Mice were subsequently instilled with PBS or influenza on day 14 post vaccination (Figure 3A). After seven days of influenza infection, aged mice were intranasally instilled with *S. pneumoniae*, and lung tissue was collected at 24 h post-secondary infection (Figure 3A). To examine if Pneumovax treatment altered cellular recruitment or viremia, total cell numbers and viral titers in BAL isolated from PBS- and Pneumovax treated- mice were quantified (Figure 3B,C). When compared to PBS controls, there was no significant increase in cellular recruitment or viral titers in aged adult mice vaccinated with Pneumovax on day 7 post influenza (Figure 3B,C). To determine if vaccination with Pneumovax resulted in IgM and IgG antibody titers, we collected serum from PBS- and Pneumovax-treated mice at select time points post immunization and quantified IgM and IgG titers against pneumococcal polysaccharide serotype 3 (PPS3) [33]. There was a detectable increase in a-PPS3 IgM and IgG antibodies detected in aged adult serum post vaccination (Figure 3 D,E). Based on these findings, we next examined if treatment with Pneumovax altered cellular recruitment during primary and secondary *S. pneumoniae* infection. As shown in Figure 3F, when compared to primary infection, there was a significant increase in total cell numbers during a secondary, post-viral *S. pneumoniae* infection. Despite this increase, there was no significant difference in cells present in BAL collected from PBS- and Pneumovax-treated aged adult mice (Figure 3F). In response to influenza infection, there was a significant increase in bacterial titers present in aged lung (Figure 3G). Secondary *S. pneumoniae* titers in aged mice vaccinated with Pneumovax were significantly lower than the titers observed in unvaccinated mice (Figure 3G). When compared to aged mice receiving only a primary infection with *S. pneumoniae*, the presence of influenza significantly decreased vaccine efficacy and resulted in augmented *S. pneumoniae* titers in aged lung (Figure 3G). As protein-rich edema can interact with alveolar surfactants and result in decreased pulmonary compliance, we next examined if there were changes in protein levels present in BAL collected from control and infected aged mice. Despite vaccination, there was increased BAL protein in aged lung in response to influenza, which was significantly heightened in response to secondary *S. pneumoniae* (Figure 3H). Taken together, our results illustrate that influenza contributes to decreased bacterial clearance and increased lung injury in both untreated and Pneumovax-vaccinated mice.

### 3.4. Influenza-Mediated Changes in IL1β and IL6 Cytokine Production in Pneumovax Vaccinated Aged Adult Mice during S. pneumoniae Infection

**Figure 4:**
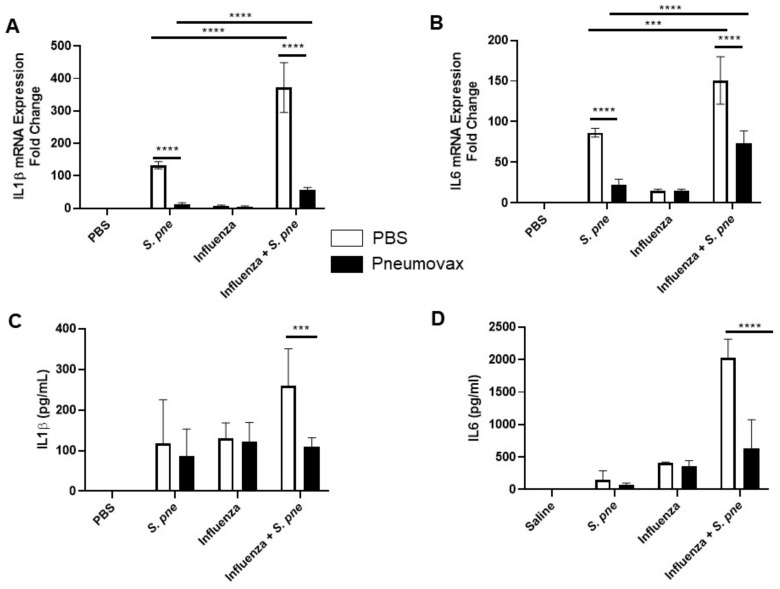
Influenza-mediated changes in IL-1β and IL-6 cytokine production in Pneumovax-vaccinated aged adult mice during *S. pneumoniae* infection. Aged adult (18–20 months) mice received a subcutaneous injection of PBS or Pneumovax (5 mg/mouse) on day 0. On day 14 post vaccination, mice were intranasally instilled with PBS or influenza (A/Puerto Rico/8/1934, H1N1) prior to secondary intranasal instillation with *S. pneumoniae* (1 × 10^3^ CFU) on day 7 post influenza. (**A**) IL-1β and (**B**) IL-6 mRNA expression was assessed in lung tissue collected from unvaccinated and Pneumovax-treated mice at 24 h post *S. pneumoniae*. (**C**) IL-1β and (**D**) IL-6 cytokine production was quantified by ELISA using BAL collected from unvaccinated and Pneumovax-treated mice at 24 h post *S. pneumoniae*. Unvaccinated and Pneumovax mice receiving only PBS or influenza alone were used as controls. Student’s *t*-test: *** *p* < 0.001 and **** *p* < 0.0001. Similar results were obtained from at least three independent experiments, with N = 5 per group. Data are expressed as the mean ± SD.

We next examined if a primary infection with influenza altered pro-inflammatory cytokine production in aged lung in response to *S. pneumoniae*. There was a significant increase in IL-1β and IL-6 mRNA (Figure 4A,B) as well as IL-1β and IL-6 cytokine production (Figure 4C,D) in aged lung in response to secondary post-influenza infection with *S. pneumoniae*. To investigate the impact of influenza on pneumococcal vaccine efficacy, we evaluated inflammatory cytokine production in response to primary and secondary post-influenza *S. pneumoniae* infection. In response to Pneumovax, there was a significant decrease in IL-1β and IL-6 mRNA (Figure 4A,B) as well as IL-1β and IL-6 cytokine production (Figure 4C,D) in aged lung during primary and secondary *S. pneumoniae* infection when compared to unvaccinated controls. Despite improvement in inflammatory cytokine production by Pneumovax, these outcomes were directly impacted by influenza, with significantly higher levels of IL-6 mRNA and cytokine production being detected in aged lung during secondary *S. pneumoniae* infection (Figure 4B,D). In sum, our results demonstrate that there is augmented IL-1β and IL-6 production in aged lung in response to a post-influenza infection with *S. pneumoniae*. While these levels are decreased in response to Pneumovax vaccination, they remained significantly elevated in post-influenza *S. pneumoniae*-infected mice.

### 3.5. Influenza-Mediated Changes in NFҡB Signaling in Aged Adult Lung Contribute to Changes in Pneumovax Vaccine Efficacy

**Figure 5:**
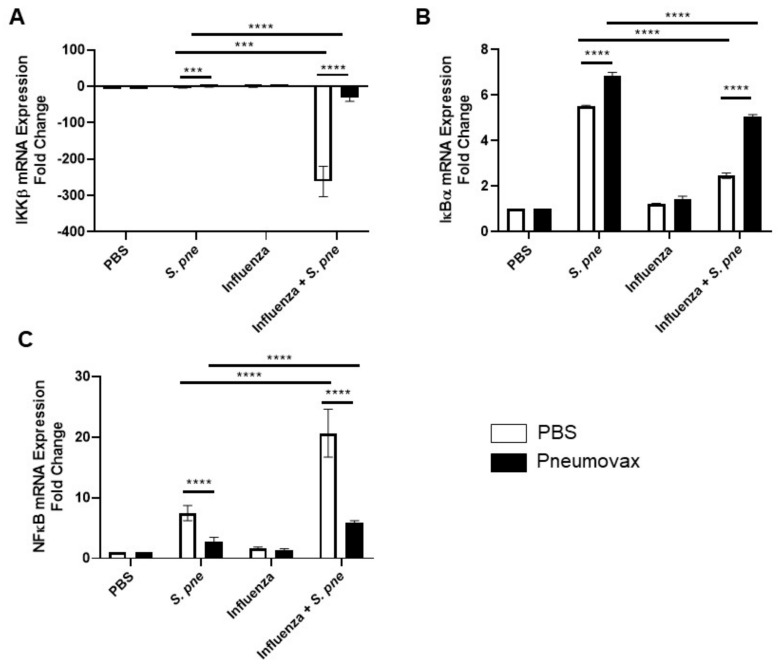
Influenza-mediated changes in NFҡB signaling in aged adult lung contribute to changes in Pneumovax vaccine efficacy. Aged adult (18–20 months) mice received a subcutaneous injection of PBS or Pneumovax (5 mg/mouse) on day 0. On day 14 post vaccination, mice were intranasally instilled with PBS or influenza (A/Puerto Rico/8/1934, H1N1) prior to secondary intranasal instillation with *S. pneumoniae* (1 × 10^3^ CFU) on day 7 post influenza. (**A**) IKKβ, (**B**) IҡBα, and (**C**) NFҡB mRNA expression was assessed in lung tissue collected from unvaccinated and Pneumovax-treated mice at 24 h post *S. pneumoniae*. Unvaccinated and Pneumovax mice receiving only PBS or influenza alone were used as controls. Student’s *t*-test: *** *p* < 0.001 and **** *p* < 0.0001. Similar results were obtained from at least three independent experiments, with N = 5 per group. Data are expressed as the mean ± SD.

As IL-6 production is controlled by changes in NFҡB signaling, we next investigated the impact of influenza on the expression of several components of the NFҡB pathway. We first examined the impact of primary influenza infection on changes in the expression pattern of the NFҡB inhibitor IKKβ. There was a significant decrease in IKKβ mRNA expression in aged lung during a secondary post-influenza infection with *S. pneumoniae* (Figure 5A). Vaccination with Pneumovax increased IKKβ mRNA expression during primary and secondary *S. pneumoniae* infection (Figure 5A). Despite an improvement with Pneumovax vaccination, when compared to primary *S. pneumoniae*, IKKβ mRNA expression in aged lung remained significantly lower during a secondary post-influenza infection (Figure 5A). We next evaluated the impact of a primary infection with influenza on the expression of NFҡB inhibitor IҡBα. There was a significant decrease in IҡBα mRNA expression in aged lung during a secondary post-influenza infection with *S. pneumoniae* (Figure 5B). Vaccination with Pneumovax increased IҡBα mRNA expression during both primary and secondary *S. pneumoniae* infections (Figure 5B). Despite an improvement with Pneumovax vaccination, when compared to primary *S. pneumoniae*, IҡBα mRNA expression in aged lung remained significantly lower during a secondary post-influenza infection (Figure 5B). Based on these findings, we next examined the impact of influenza on NFҡB mRNA expression. There was a significant increase in NFҡB mRNA expression in aged lung during a secondary post-influenza infection with *S. pneumoniae* (Figure 5C). Vaccination with Pneumovax decreased NFҡB mRNA expression during both primary and secondary *S. pneumoniae* infections (Figure 5C). Despite an improvement with Pneumovax vaccination, when compared to primary *S. pneumoniae*, NFҡB mRNA expression in aged lung remained significantly elevated during a secondary post-influenza infection (Figure 5C). Taken together, our results illustrate that, in response to influenza, there was an increase in NFҡB gene expression detected in aged lung during secondary *S. pneumoniae* infection. Vaccination with Pneumovax improved the expression of NFҡB inhibitors, IKKβ and IҡBα; however, these levels were decreased when compared to expression levels in the aged lung during primary *S. pneumoniae* infection.

## 4. Discussion

The purpose of our current study was to investigate the role of immune imprinting by influenza on pneumococcal vaccine effectiveness during *Streptococcus pneumoniae* infection in the aged murine lung. Our results illustrate that, in response to a primary influenza infection, there was diminished bacterial clearance and heightened production of pro-inflammatory cytokines, such as IL-6 and IL-1β. Vaccination with Pneumovax decreased pro-inflammatory cytokine production by modulating NF-ҡB expression; however, these responses were significantly diminished after influenza infection. Taken together, the data in our current study illustrate that immune imprinting by influenza diminishes pneumococcal vaccine efficacy and, thereby, may contribute to increased susceptibility of older persons to a secondary infection with *S. pneumoniae*.

For our studies, we chose to use the FDA-cleared vaccine Pneumovax to examine the impact of influenza on vaccine efficacy to secondary *S. pneumoniae* infection. For adults >65 years of age, the 23-valent pneumococcal polysaccharide vaccine (PPV23) Pneumovax and the 13-valent pneumococcal conjugate vaccine (PCV13) Prevnar are two vaccines available for protection against pneumococcal infections. While there have been conflicts in pneumococcal vaccine effectiveness, a recent meta-analysis illustrated that Pneumovax exhibited a weak protective effect on all-cause pneumonia among immunocompetent adults and persons over 65 years of age as well as high-risk persons (19–64 years of age) [34]. While multiple studies have illustrated that vaccination in persons >60 years with Prevnar can result in improved immunogenicity against multiple *S. pneumoniae* serotypes, these antibody titers were found to decline after a year and were similar to titers observed post Pneumovax vaccination [35,36,37,38]. In addition, combined administration of Prevnar prior to Pneumovax can elicit a greater immune response than multiple dosages of Prevnar, which only demonstrated a modest increase [37,39]. As recent work has illustrated differential efficacy of Prevnar vaccination in modulating the immune responses of adult mice to a post-influenza infection with a serotype 3 strain of *Streptococcus pneumoniae*, we chose to examine the impact of Pneumovax on these responses [30,40]. Given these findings, it would be plausible that other vaccines, designed with specific bacterial components, such as pneumococcal surface protein A (PspA) and pneumolysin (Ply) or liposomal encapsulation of polysaccharides (LEPS), may improve efficacy to post-influenza *S. pneumoniae* infection [31,43,44,45]. Future work will need to evaluate if additional vaccine types can prove efficacious and further reduce inflammatory cytokine production while improving bacterial clearance post-influenza infection.

In response to Pneumovax, in agreement with previous studies, we detected PPS3-specific IgM and IgG in serum collected from aged adult mice by day 14 post vaccination [33,46]. Based on these findings, we chose to examine the impact of influenza on modulating vaccine efficacy against a secondary pneumococcal infection. It is important to note that, at day 14 post vaccination, the effector phase of the adaptive immune response was not completely cleared after vaccine administration. While we observed efficacy of Pneumovax vaccination in decreasing the production of proinflammatory cytokines, such as IL-6, and decreased bacterial titers during secondary *S. pneumoniae* infection, an increased duration of time after vaccination, when all effector immune cells have rested into a memory phenotype, may further improve these responses.

Neuraminidases (NA) are glycoside hydrolase enzymes that, through the cleavage of glycosidic linkages, can facilitate the mobility of virus particles through the respiratory tract mucus as well as aid in viral elution from infected cells. Influenza viral NA has been previously shown to be an important factor in viral-bacterial synergism [24,29]. Increased sialic acid cleavage by viral NA can promote greater pneumococcal adherence and bacterial invasion [47,48]. This synergistic relationship can aid in successful invasion of the lower respiratory tract by *S. pneumoniae* during influenza infection [48]. Of note, *S. pneumoniae* NA, which plays an important role in biofilm formation, can also help facilitate pneumococcal pathogenesis and respiratory tract colonization [49]. Given the properties of viral and bacterial neuraminidases, it is possible that the increased bacterial burden detected in Pneumovax-treated lung after secondary *S. pneumoniae* infection was due to a greater ability of the bacteria to bind to the respiratory epithelium. Despite bacterial suppression from the antibody response, increased sialic acid cleavage by influenza NA during the primary infection can contribute to augmented bacterial titers during a secondary infection with *S. pneumoniae* when compared to Pneumovax-treated lung during a primary *S. pneumoniae* infection alone.

Influenza virus, respiratory syncytial virus, parainfluenza virus, adenovirus, and coronavirus are commonly detected in patients with community-acquired pneumonia. Unfortunately, at present, it is not fully clear to what extent these pathogens contribute to disease development or predispose a patient to secondary infections [50,51]. While our work has focused on influenza, additional viral pathogens have the propensity to alter vaccine efficacy. It is therefore plausible that, while our results illustrate a specific mechanism by which inflammatory cytokine production is altered in response to influenza, similar inhibitory mechanisms might underlie decreased vaccine efficacy against secondary pneumococcal infections. Future studies will need to be performed to examine if altered NFҡB signaling is a common mechanism that underlies increased inflammation during secondary post-viral pneumococcal infections.

It is important to note that the timing between influenza infection and post-viral pneumococcal infection as well as the dose of bacteria administered can greatly impact the host outcomes to a secondary infection [17,20,21,22,23]. Initiation of influenza-virus-mediated lung inflammation has been shown to directly correlate with bacterial growth, and high dosages of bacteria were associated with increased mortality during secondary infection [17,20,21,22,23]. In agreement with previous studies, results from our current work further illustrated that, as early as day 3 post influenza, bacterial clearance from lung was significantly altered in young adult lung, with significantly increased titers present in aged adult lung [25]. Influenza-mediated changes in the immune response contributed to increased virus-mediated damage to the lung and heightened susceptibility to secondary pneumococcal infection [18,26,27,28]. In our model of influenza and secondary *S. pneumoniae* infection, we detected a similar phenotype that occurred by day 7 of influenza, with more extensive damage observed in the aged adult lung. These results extend the findings from our previous work, which illustrated that an age-associated decrease in NLRP3 inflammasome activation contributed to increased morbidity and mortality of aged adult mice to secondary *S. pneumoniae* at day 14 post influenza [52]. Similar to previously published studies in young adult mice, we also observed a dramatic reduction in vaccine efficacy against *S. pneumoniae* in the presence of influenza [17,30,31]. Based on these results, it is possible that the immune priming response that occurs during influenza can have a direct negative impact on adaptive vaccine responses in aged adult lung. Future work will need to be performed to evaluate if antibody titers can directly modulate host–pathogen interactions.

Previous work has illustrated that there is a relationship between the lung permeability index (LPI) and total protein measured in the bronchoalveolar lavage, with increased protein leak into the lungs being indicative of a continuing event or injury [53]. Results of our current study illustrate that, despite vaccination, there was increased protein-rich edema present in the aged lung in response to influenza that was significantly heightened in response to secondary *S. pneumoniae.* It is possible that, despite pneumococcal vaccination, extensive viral-mediated damage to the epithelium by influenza can augment protein leakage into the lungs. It is important to note that, while we examined protein levels in BAL fluid collected from PBS- and Pneumovax-treated mice during primary and secondary *S. pneumoniae* infection, there are multiple types of protein and lipids present within each BAL sample. Pulmonary surfactant is a complex mixture of lipids and proteins that consists of lipids (90%) and proteins (10%) [54]. Both hydrophilic surfactant protein (SP), SP-A and SP-D, and hydrophobic proteins, SP-B and SP-C, play important roles in modulating innate immune responses at the alveolar barrier and maintaining the biophysical activity of pulmonary surfactant [54,55]. In the current study, we focus on a potential mechanistic pathway that may underlie increased susceptibility of aged lung to secondary *S. pneumoniae* infection. As SP-B has been previously shown to play an important role in preventing respiratory failure during acute lung injury, future work will need to be performed to examine the exact surfactant protein composition of BAL collected during primary and secondary *S. pneumoniae* and the impact of pneumococcal vaccination on these protein concentrations [56,57].

In summary, the results of our current study illustrate that immune imprinting by influenza diminishes pneumococcal vaccine efficacy and, thereby, may contribute to increased susceptibility of older persons to a secondary infection with *S. pneumoniae*.

## 5. Conclusions

- Primary influenza infection increases susceptibility of aged adult mice to *S. pneumoniae*.
- Primary influenza infection impairs pneumococcal vaccine effectiveness during *S. pneumoniae* infection in aged lung
- Influenza-mediated changes in NFκB signaling in aged adult lung contribute to changes in pneumococcal vaccine efficacy.
